# Induction of therapeutic immunity and cancer eradication through biofunctionalized liposome-like nanovesicles derived from irradiated-cancer cells

**DOI:** 10.1186/s12951-024-02413-8

**Published:** 2024-04-08

**Authors:** Suke Deng, Jiacheng Wang, Yan Hu, Yajie Sun, Xiao Yang, Bin Zhang, Yue Deng, Wenwen Wei, Zhanjie Zhang, Lu Wen, You Qin, Fang Huang, Yuhan Sheng, Chao Wan, Kunyu Yang

**Affiliations:** 1 Cancer Center, Union Hospital, Tongji Medical College, Huazhong University of Science and Technology, Wuhan, 430022 China; 2 Institute of Radiation Oncology, Union Hospital, Tongji Medical College, Huazhong University of Science and Technology, Wuhan, 430022 China; 3 Hubei Key Laboratory of Precision Radiation Oncology, Wuhan, China

**Keywords:** Liposome-like nanovesicle, Cancer immunotherapy, Tumor-associated macrophages, Tumor microenvironment

## Abstract

Immunotherapy has revolutionized the treatment of cancer. However, its efficacy remains to be optimized. There are at least two major challenges in effectively eradicating cancer cells by immunotherapy. Firstly, cancer cells evade immune cell killing by down-regulating cell surface immune sensors. Secondly, immune cell dysfunction impairs their ability to execute anti-cancer functions. Radiotherapy, one of the cornerstones of cancer treatment, has the potential to enhance the immunogenicity of cancer cells and trigger an anti-tumor immune response. Inspired by this, we fabricate biofunctionalized liposome-like nanovesicles (BLNs) by exposing irradiated-cancer cells to ethanol, of which ethanol serves as a surfactant, inducing cancer cells pyroptosis-like cell death and facilitating nanovesicles shedding from cancer cell membrane. These BLNs are meticulously designed to disrupt both of the aforementioned mechanisms. On one hand, BLNs up-regulate the expression of calreticulin, an “eat me” signal on the surface of cancer cells, thus promoting macrophage phagocytosis of cancer cells. Additionally, BLNs are able to reprogram M2-like macrophages into an anti-cancer M1-like phenotype. Using a mouse model of malignant pleural effusion (MPE), an advanced-stage and immunotherapy-resistant cancer model, we demonstrate that BLNs significantly increase T cell infiltration and exhibit an ablative effect against MPE. When combined with PD-1 inhibitor (α-PD-1), we achieve a remarkable 63.6% cure rate (7 out of 11) among mice with MPE, while also inducing immunological memory effects. This work therefore introduces a unique strategy for overcoming immunotherapy resistance.

**Supplementary Information:**

The online version contains supplementary material available at 10.1186/s12951-024-02413-8.

## Introduction

Immunotherapy with immune-checkpoint inhibitors (ICIs) is extensively used in clinics and has enabled a leap forward in the treatment of various types of cancer in recent years. The journal Science declared cancer immunotherapy as the breakthrough of the year in 2013, based on the great success in ICIs in solid cancer and advances in engineered T cells in blood malignancies [1]. However, the efficacy of immunotherapy with ICIs on most patients is not satisfactory [2]. According to a meta-analysis, 28,304 patients from 160 studies were included, of which 4747 responses occurred in 22,165 patients treated with PD-1/PD-L1 inhibitors, the combined objective response rates (ORRs) were only 20.21% [3]. Though the mechanisms by which cancer cells resist to ICIs are complicated, most of them can be attributed to the cancer cells or the tumor microenvironment (TME) factors [4]. In numerous types of cancers, the expression of immune sensors, such as MICA, FAS, MHC-I, CD155, ULBP 2/5/6, are down-regulated, which causes disorders in the recognition of cancer cells by immune cells [5–8]. Meanwhile, immune cells, such as tumor-associated macrophages (TAMs), in the TME often polarize to an immunosuppressive phenotype which supports cancer progression and resistance to therapy [9]. Therefore, exploring new strategies that can remodel both the immunogenicity of cancer cells and the functions of immune cells will further improve the therapeutic effect of ICIs on cancer.

Radiotherapy (RT) is one of the most-effective cytotoxic therapies available for the treatment of localized solid cancers, and more than half of all patients with cancer will receive RT as part of their treatment [10]. In addition to its ability to mediate DNA-breakage induced cancer cell death, RT can enhance the immunogenicity of cancer cells, increasing tumor-infiltrating lymphocytes, which is widely summarized as turning immunologically “cold” cancers “hot”. However, RT is widely used as a local treatment of cancers. Once the cancer has metastases, the use of RT becomes limited [11–14]. In previous studies, we found that irradiated-cancer cells derived microparticles (RT-MPs) can not only kill cancer cells through ferroptosis, but also remodel TAMs to an M1-like (pro-inflammatory and usually anti-cancer) phenotype, conferring a cancer ablative effect in the MPE mouse model. These results suggested that the extracellular vesicles released by irradiated-cancer cells can play a role similar to that of RT [15]. 

Inspired by the profound effect of RT on modulating both the immunogenicity of cancers and the TME, we constructed BLNs via exposing irradiated-cancer cells to ethanol, of which ethanol served as a surfactant, inducing cancer cells pyroptosis-like cell death and facilitate the BLNs shedding from cancer cell membrane [16]. Further, we demonstrated that the BLNs showed better anti-cancer activity than RT-MPs in up-regulating the expression of calreticulin on the surface of cancer cells, while remodeling the TAMs toward the M1-like phenotype in vivo and in vitro, synergistically facilitating macrophage phagocytosis of cancer cells as well as triggering anticancer T-cell immunity. Combined with BLNs, the immunotherapy with α-PD-1 can further completely remove cancer cells in the MPE mouse model and immunological memory effects were induced.

## Results

### Rational design, preparation and characterization of BLNs

**Fig. 1:**
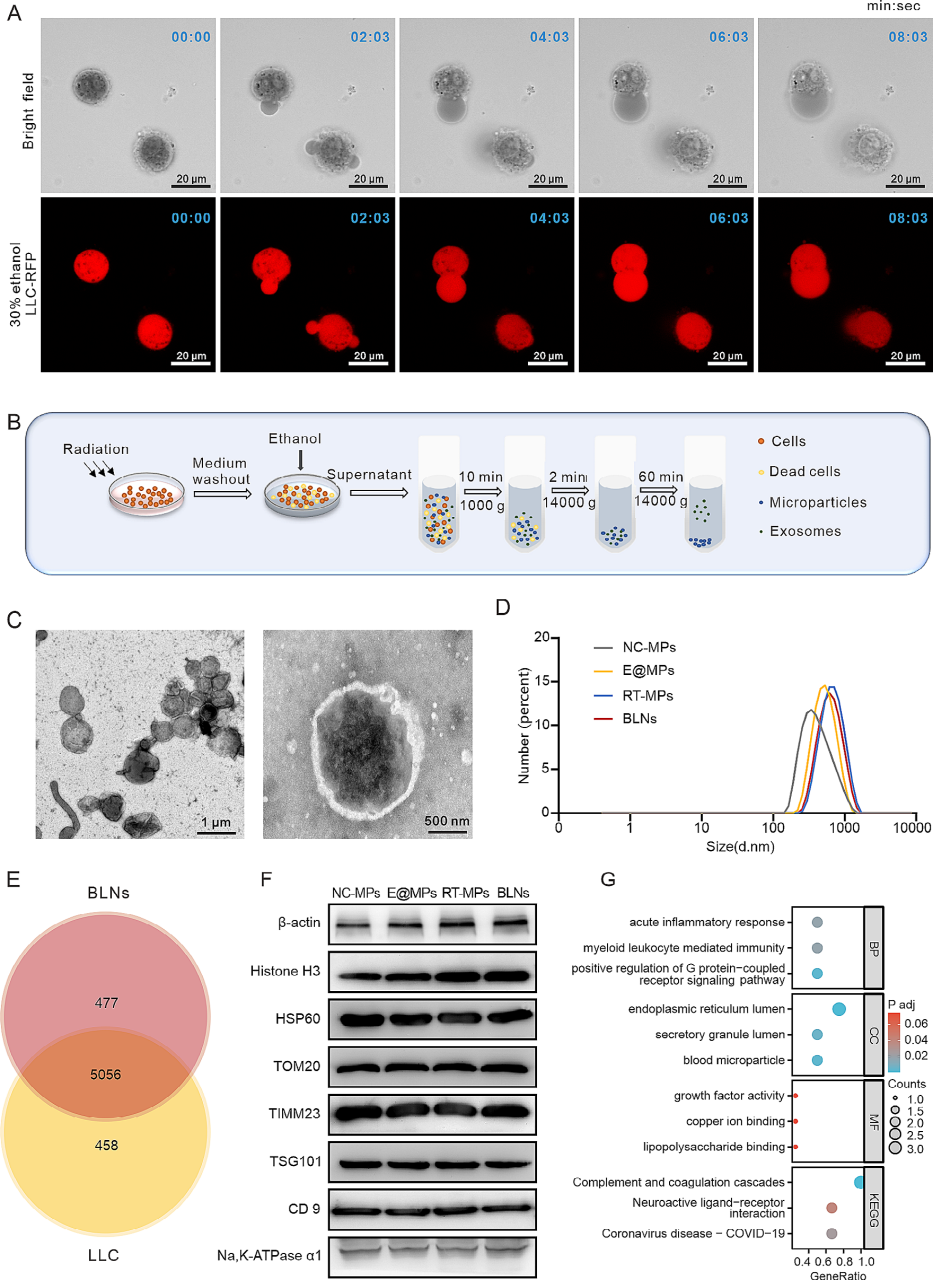
Rational design, preparation and characterization of BLNs. (**A**) Microscopy imaging of LLC-RFP released BLNs. Shown are representative time-lapse cell images (brightfield and fluorescence) taken from 0–10 min after 30% ethanol addition. Scale bar: 20 μm. (**B**) Schematic diagram for the procedure used to obtain the BLNs. (**C**) TEM image of BLNs, scale bar:1 μm (left) or 500 nm (right). (**D**) Size distribution of LLC-derived MPs, E@MPs, RT-MPs and BLNs by dynamic light scattering. (**E**) Venn diagram of the overlap proteins between LLC and BLNs. (**F**) Western blots of mitochondrial marker proteins, extracellular vesicle marker proteins, histones and β-actin expression in LLC-derived MPs, E@MPs, RT-MPs and BLNs. (**G**) KEGG enrichment analysis of top 100 proteins that were significantly enriched in BLNs compared to LLC

To maximize BLNs yield, we stimulated microparticle release through external interference. The ability of ethanol to enhance extracellular vesicle formation in hepatocytes inspired our investigation of microparticle formation in Lewis lung carcinoma cells stably transfected with red fluorescent protein (LLC-RFP) at varying ethanol concentrations [17, 18]. Different ethanol concentrations induced distinct cellular responses, with low concentrations failing to effectively release vesicles and high concentrations leading to premature cell disintegration and limited vesicle production. (Figure S1A). However, we found that 30% ethanol stimulation ensured optimum vesicle production in a way similar to pyroptosis described in the work of Shao Feng et al., without mere enlargement or sudden rupture of treated cells (Fig. 1A and Movie S1) [16]. To verify whether tumor cells undergo pyroptosis after 30% ethanol treatment, we examined the cleavage of classic pyroptosis markers highly expressed in LLC **(**Figure S1B). Western blot showed that 30% ethanol stimulation did not affect the cleavage of GSDMD and GSDME **(**Figure S1C), which means the 30% ethanol treatment only induced a morphological change similar to pyroptosis, referring to as pyroptosis-like cell death. Considering that alcohol may affect the function of proteins on the membrane of BLNs, we explored the effect of 30% alcohol on the function of membrane proteins, and we chose calreticulin (CRT) as a representative to study. We found that 30% alcohol did not attenuate the function of CRT on macrophage activation (Figure S1D, E).

Previously, we observed that exposure to high-dose radiation (20 Gy) could trigger cancer cells to release tumoricidal RT-MPs. Therefore, we prepared irradiated cancer cells with 30% ethanol and extracted BLNs through gradient centrifugation from the supernatant (Fig. 1B). Transmission electron microscopy (TEM) showed that the particles had a spherical structure with a slightly undulated surface and measured between 500 and 700 nm in diameter (Fig. 1C). Subsequently, we used Malvern Particle-sizer to measure the size of spontaneously produced MPs (N-MPs), MPs stimulated only with 30% ethanol (E@MPs), RT-MPs, and BLNs. The mean sizes of these particles were 342, 531, 681.5, and 615 nm, respectively (Fig. 1D). Flow Nano-Analyzer confirmed a higher concentration of vesicles of BLNs (Figure S1F). Extracellular-vesicles (EVs) are known to contain a variety of bioactive molecules. To explore the protein content of BLNs, proteomic profiling exhibited that 90% of the proteins detected in BLNs overlapped with those in parental cancer cells (Fig. 1E). In addition to typical EV-associated proteins such as NA, K-ATPase α1, CD9, and tumor susceptibility gene 101 protein (TSG101), [19] our western blot confirmed the presence of representative cytoskeleton protein (β-actin), nuclear protein (histone H3), and mitochondrial-associated proteins including HSP60, TOM20, and TIMM23 (Fig. 1F). We selected the top 100 proteins that were significantly enriched in BLNs compared to parental cancer cells (LLC cell line) and ran KEGG enrichment analysis, which suggested that proteins in BLNs could participate in a range of cellular activities, particularly pathways involved in activating innate immunity (Fig. 1G). These findings demonstrated that we successfully synthesized a new type of tumor cell-derived microparticles, BLNs, which are rich in bioactive molecules and warrant further research in the field of tumor therapy.

### BLNs exert promising MPE therapeutic potential while evoking anti-tumor immunity

**Fig. 2:**
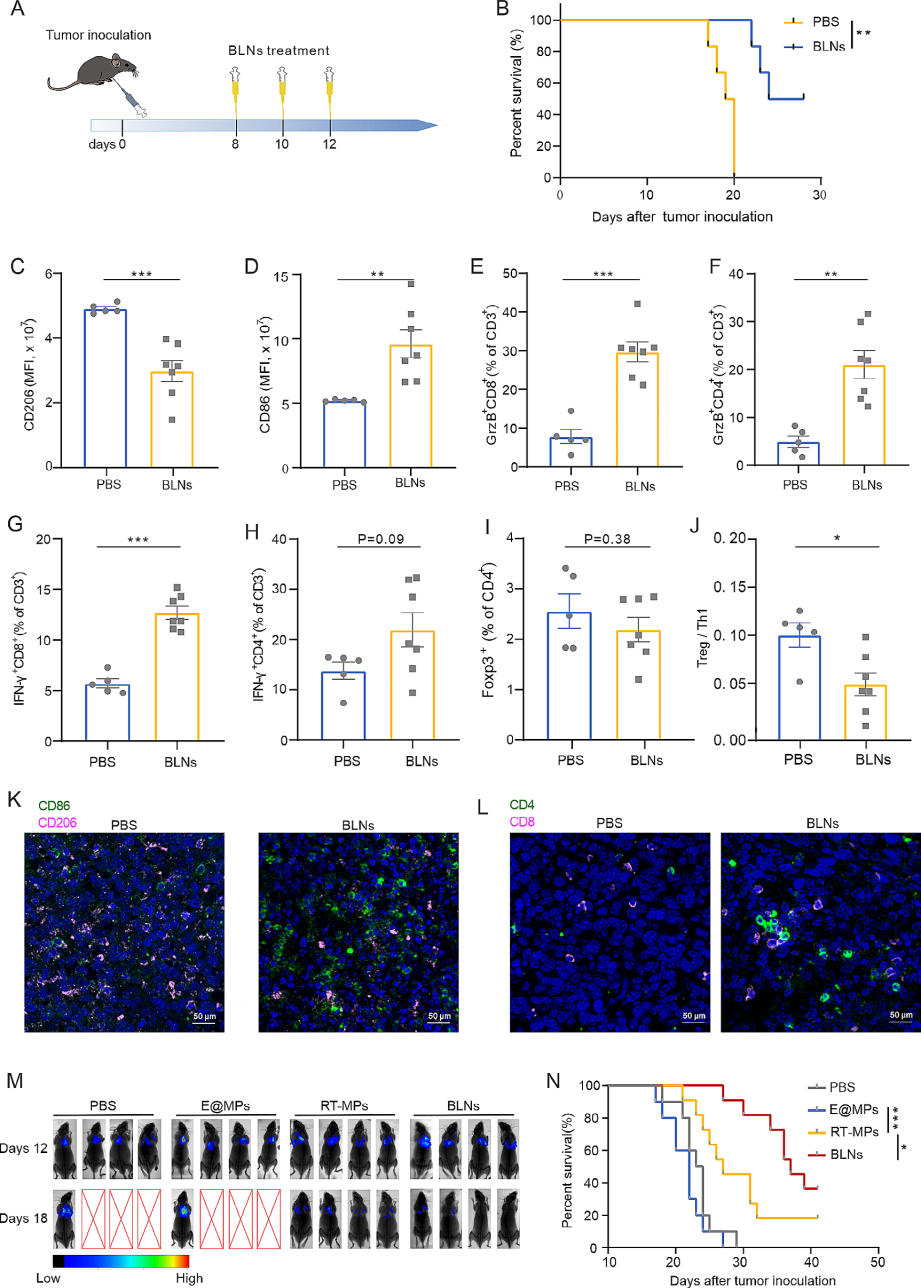
BLNs exert promising MPE therapeutic potential while evoking anti-tumor immunity. (**A**) Experimental outline of model establishment time and drug injection time. (**B**) Survival analysis between PBS- or BLNs-treated group (*n* = 10 per group). **P < 0.01. (**C-J**) Flow cytometry analysis of the changes of immune microenvironment after BLNs treatment. (**K, L**) Immunofluorescent staining of (**K**) CD86 and CD206 or (**L**) CD4 and CD8 was performed in tumor tissues after PBS or BLNs treatment. Images were obtained by confocal microscopy. Red-CD8/CD206, Green‐CD4/CD86, Blue‐DAPI. Scale bar: 20 μm. (**M**) Representative in vivo bioluminescence images showing the growth of mice MPE after various treatments. (**N**) Kaplan-Meier survival plot of MPE mice under various treatments (*n* = 9 to 10 per group)

To test the potential of BLNs as a therapeutic agent for treating cancers, we used a mouse model of MPE, an advanced-stage and immunotherapy-resistant cancer model, which usually resist to multiple types of treatment [20, 21]. We administered three doses of BLNs every other day beginning on day 8 after cancer inoculation (Fig. 2A). Significantly, BLNs treatment led to better survival in MPE mice (Fig. 2B). To further clarify the effects of BLNs on the TME in vivo, we collected both cancer nodules for cytokine analysis via ELISA and pleural effusion cells for flow cytometry. The ELISA test showed the levels of IFN-γ and IL-2 increased in cancer nodules (Figure S2A, B), indicating that anti-tumor immunity had been activated. Correspondingly, flow cytometry analysis and immunohistochemistry staining of pleural cancer nodules both demonstrated an increase in the CD86^+^ subset and a decrease in CD206^+^ subset (Fig. 2C, D, K and Figure S2C-E). Simultaneous increases in the percentages of IFN-γ^+^ or GrzB^+^ T cells of both the CD8^+^ and CD4^+^ subsets were observed as well, indicating tumoricidal T cells infiltration was prompted (Fig. 2E–H, L and Figure S2F-K). Although there was no significant difference in the Foxp3^+^ subset of CD4^+^ T cells (Fig. 2I), the Treg (Foxp3^+^CD4^+^) / Th1 (IFN-γ^+^CD4^+^) ratio decreased (Fig. 2J), indicating that the immunosuppressive forces in the TME had been weakened to some extent. Furthermore, we compared the efficacy of BLNs to that of E@MPs and RT-MPs. Survival analysis and in vivo imaging revealed BLNs to be the most effective therapy, with the highest survival rate (Fig. 2M, N). These findings suggest that BLN is a promising therapeutic agent for MPE treatment and has the potential to modulate anti-tumor immunity.

### BLNs elicit immunogenic death of cancer cells

**Fig. 3:**
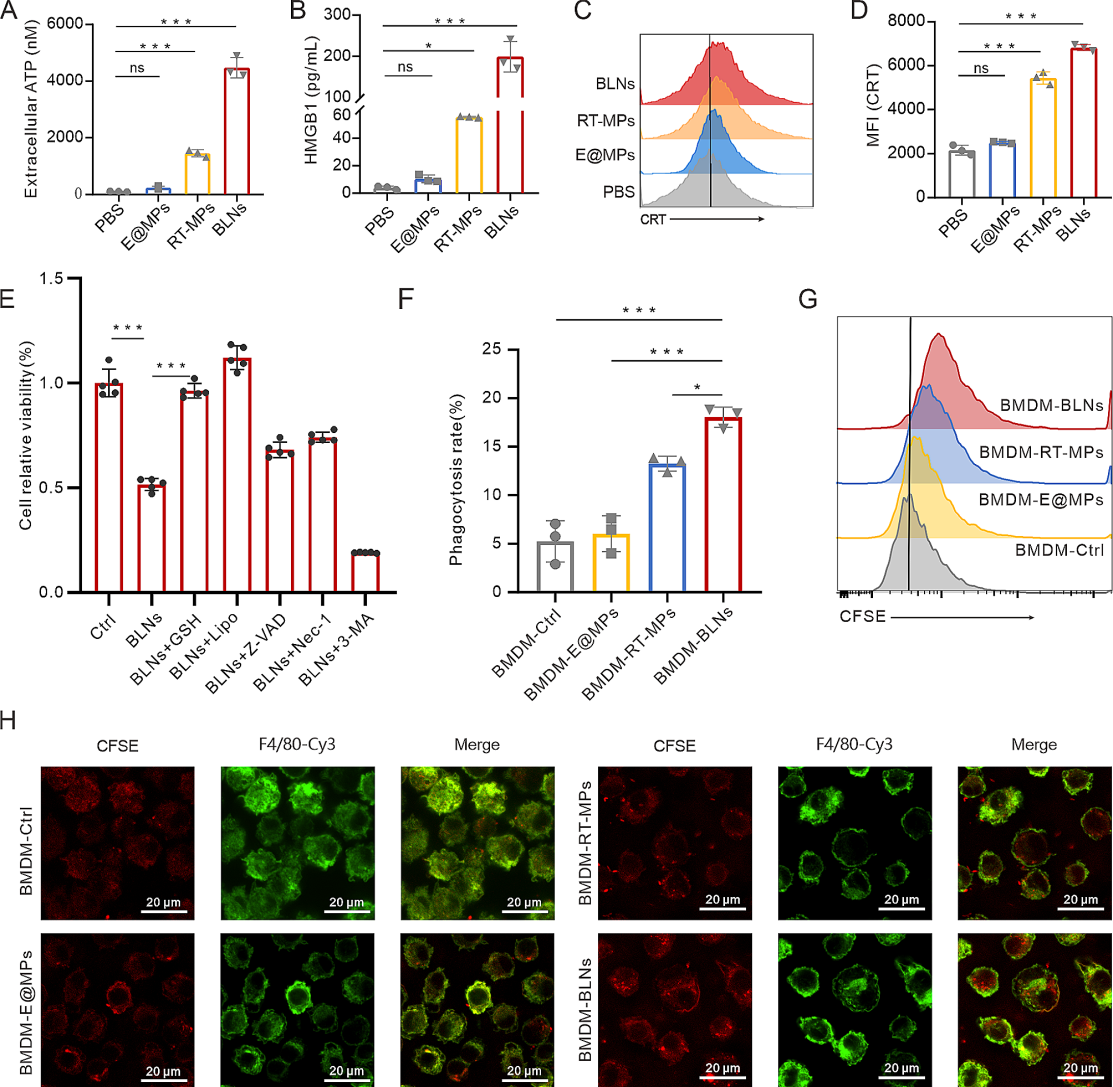
BLNs elicit immunogenic death of tumor cells. (**A**) ATP levels in LLC cells treated with PBS, E@MPs, RT-MPs, or BLNs. (**B**) HMGB1 release in LLC cells subjected to PBS, E@MPs, RT-MPs, or BLNs. (**C**) Representative flow cytometric histograms demonstrate the surface expression of CRT on LLC cells after treatment with PBS, E@MPs, RT-MPs, or BLNs. (**D**) Relative fluorescence intensity of CRT after various treatments. (**E**) Assessment of BLNs-induced cell death modalities via cck-8 assay with glutathione and cell death inhibitors. (**F**) Flow cytometry analysis of the phagocytic function of BMDMs treated with E@MPs, RT-MPs, or BLNs. (**G**) Representative flow cytometry histograms depicting the phagocytosis rates of LLC cells by BMDMs after treatment with E@MPs, RT-MPs, or BLNs. (**H**) Representative confocal images of phagocytic function of BLNs-treated BMDMs. Scale bars, 20 μm

To elucidate the mechanisms behind the therapeutic efficacy of BLNs, we first examined the effect of BLNs on cancer cells. Given that BLNs evoked a strong immune response, we hypothesized that BLNs-mediated cancer cell death created an immunogenic TME. Thus, we examined the expression of certain damage-associated molecular patterns (DAMPs) in LLC after BLNs treatment [22]. Results showed that the release of adenosine triphosphate (ATP) and high-mobility group box 1 (HMGB1), and the expression level of extracellular calreticulin (CRT) in BLNs-treated cancer cells were significantly higher than those in the control (Fig. 3A-D). As we optimized BLNs from RT-MPs, it significantly increased the expression of DAMPs compared to other types of membrane particles including RT-MPs and E@MPs. We further examined the ROS level within tumor cells post vesicle treatment and the CRT expression on indicated vesicles to clarify their differences in inducing Oxidative Stress and activating the tumor immune microenvironment. While BLNs induced a similar increase of ROS in LLC compared with RT-MPs (Figure S3A), CRT expression on BLNs surface outran RT-MPs, implying a stronger capacity to tune on TME (Figure S3B). To further explore the factors of BLNs to induce cell death, GSH and indicated cell death inhibitors were utilized to determine the mode of BLNs induced cell death. The results suggest that ferroptosis is the predominant mode of cell death induced by BLNs, although apoptosis also occurs to a lesser extent (Fig. 3E).

Immunogenic cell death of cancer cells released “find me” and “eat me” signals, which in turn promote the recruitment of phagocytic immune cells and the removal of cancer cells [23]. Therefore, we examined the phagocytosis of BLNs-treated LLC cells by bone marrow-derived macrophages (BMDMs). As expected, confocal images and flow cytometry showed that BMDMs were more likely to ingest BLNs-treated cancer cells, with a 1.2-fold increase compared to LLC treated with RT-MPs (Figure S3C-E). Furthermore, TAMs are inevitably affected by BLNs as well, so we directly tested the phagocytic ability of BMDMs treated with BLNs and found that they were better at engulfing LLC cells (Fig. 3F-H). In conclusion, BLNs induce immunogenic death of tumor cells and promote scavengers in the TME to eradicate them.

### BLNs reprogram TAMs through activating MAPK signaling pathway

**Fig. 4:**
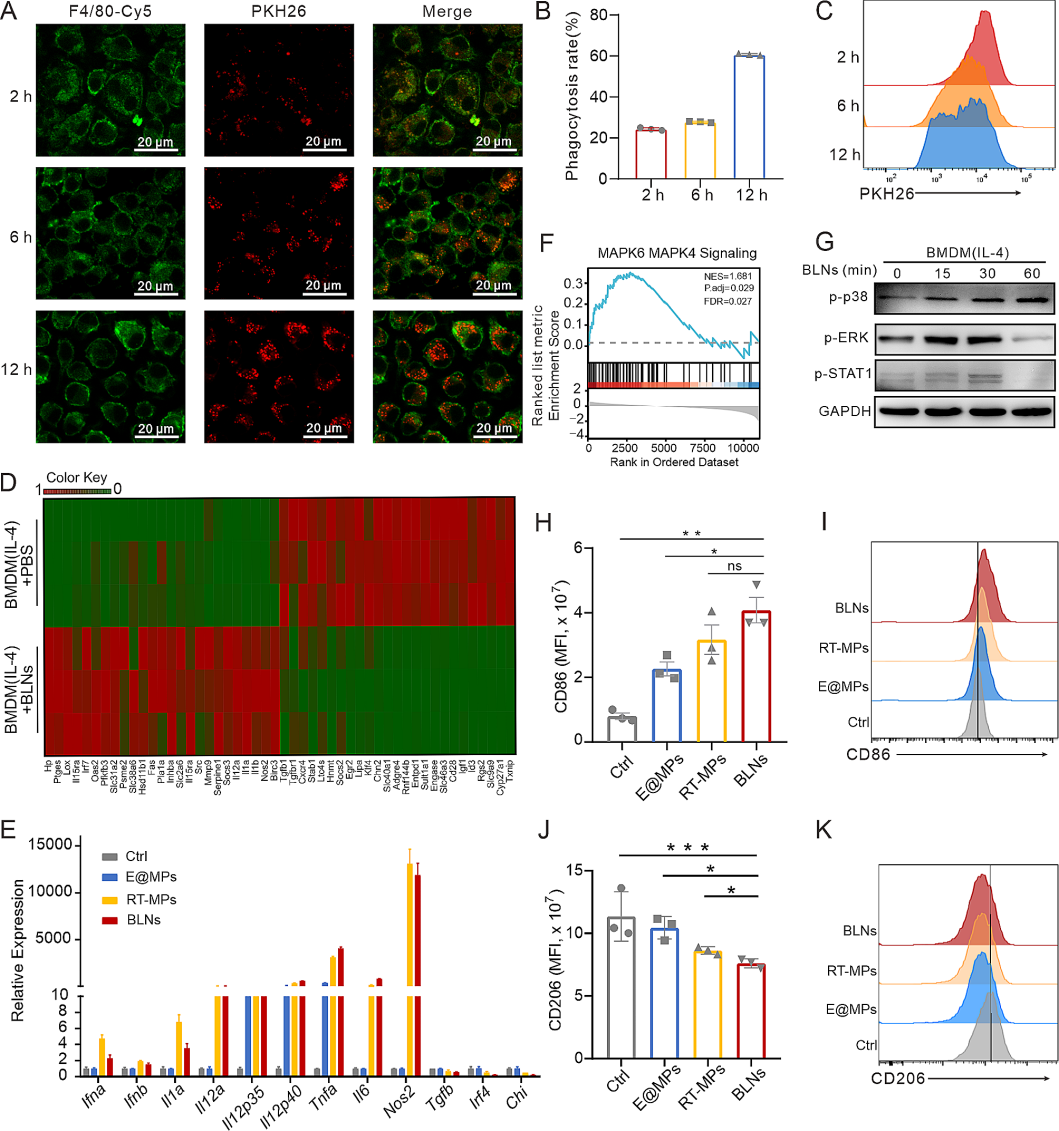
BLNs reprogram tumor-associated macrophages through activating MAPK signaling pathway. (**A**) Representative images of BLNs uptake in BMDMs at different time points. Scale bar: 20 μm. (**B, C**) Relative fluorescence intensities of internalization BLNs by macrophages at multiple time points (*n* = 3). (**D**) Heat map illustrating the differentially expressed M1-and M2‐related genes in TAMs in the BLNs group and the control group based on RNA sequencing results. (**E**) RT-qPCR analysis of the expression levels of M1- and M2-associated mRNAs in BLNs-treated BMDM-M2 cells. (**F**) Gene Set Enrichment Analysis (GSEA) plot and gene sets for MAPK6/MAPK4. (**G**) Representative western blot images of p-p38, p-ERK, p-STAT1, GAPDH in BMDM-M2 cells treated with BLNs at the different time points.** (H, J) **Representative expression of CD206 and CD86 in BMDMs after incubation with different EVs .** (I, K)** Relative fluorescence intensity of CD206 and CD86 in BMDMs after incubation with different EVs by flow cytometry

The above results suggest that BLNs can directly activate TAMs and enhance their phagocytic activity. We therefore set out to investigate the mechanisms underlying such a phenomenon. First, we examined the uptake of PKH26-labeled BLNs by macrophages. Confocal images and flow cytometry showed that BLNs were gradually absorbed by macrophages over 12 h (Fig. 4A-C). RNA-seq analysis of IL-4 induced BMDM-M2 cells treated with BLNs revealed a significant upregulation of genes related to M1 polarization (Nos2, Il1a, Il1b, Il12a), which was further validated through real-time quantitative PCR (RT-qPCR) (Fig. 4D, E). Elisa test of the supernatant from BLNs treated M2 macrophages also revealed an increase of IL-1α and IL-12 secretion, along with a decrease of TGF-β production (Figure S4A). Western blot further verified the upregulation of iNOS and downregulation of IRF4 (Figure S4B). Enrichment analysis by GSEA revealed that the MAPK6/MAPK4 signaling pathway was one of the most significantly enriched pathways after treatment with BLNs (Fig. 4F, Figure S4C-G), which is crucial for M1 polarization [24]. Subsequent western blot analysis also confirmed an increase in the phosphorylation levels of p38, ERK, and Stat1 following BLNs stimulation, suggesting the activation of MAPK signaling and its downstream transcriptional regulation (Fig. 4G). Consistently, compared to other membrane particles, BLNs were found to be the most effective in upregulating CD86 and downregulating CD206 expression on BMDMs, confirming that macrophages were polarized towards an M1 phenotype (Fig. 4H-K). In order to exclude the possible deviation caused by the method of BMDM induction, we further compared the effects of IL-4 alone or the combination of IL-10 with IL-4 to induce BMDMs into M2 phenotype. It was found that both methods could effectively induce M2 macrophages, which could be reprogrammed by BLNs to M1 phenotype (Figure S4H-J). As we observed the different ability of indicated vesicles to repolarize M2 macrophages, we next explore the mechanism behind. Noting that vesicles associated with radiation treatment (BLNs, RT-MPs) possess higher histone content than those from non-radiated counterparts (N-MPs and E@MPs) (Fig. 1F), suggesting a potential role of the dsDNA components carried by the radiation-associated vesicles. To test this hypothesis, we depleted dsDNA from BLNs, RT-MPs, and E@MPs and found that the removal of dsDNA diminished the reprogramming capacity of RT-MPs and BLNs (Figure S4K). In summary, our finding suggest that TAMs phagocytose BLNs and subsequently polarize towards an M1 phenotype upon MAPK signaling activation.

### Combination of BLNs and α-PD-1 significantly activates anti-tumor immunity

**Fig. 5:**
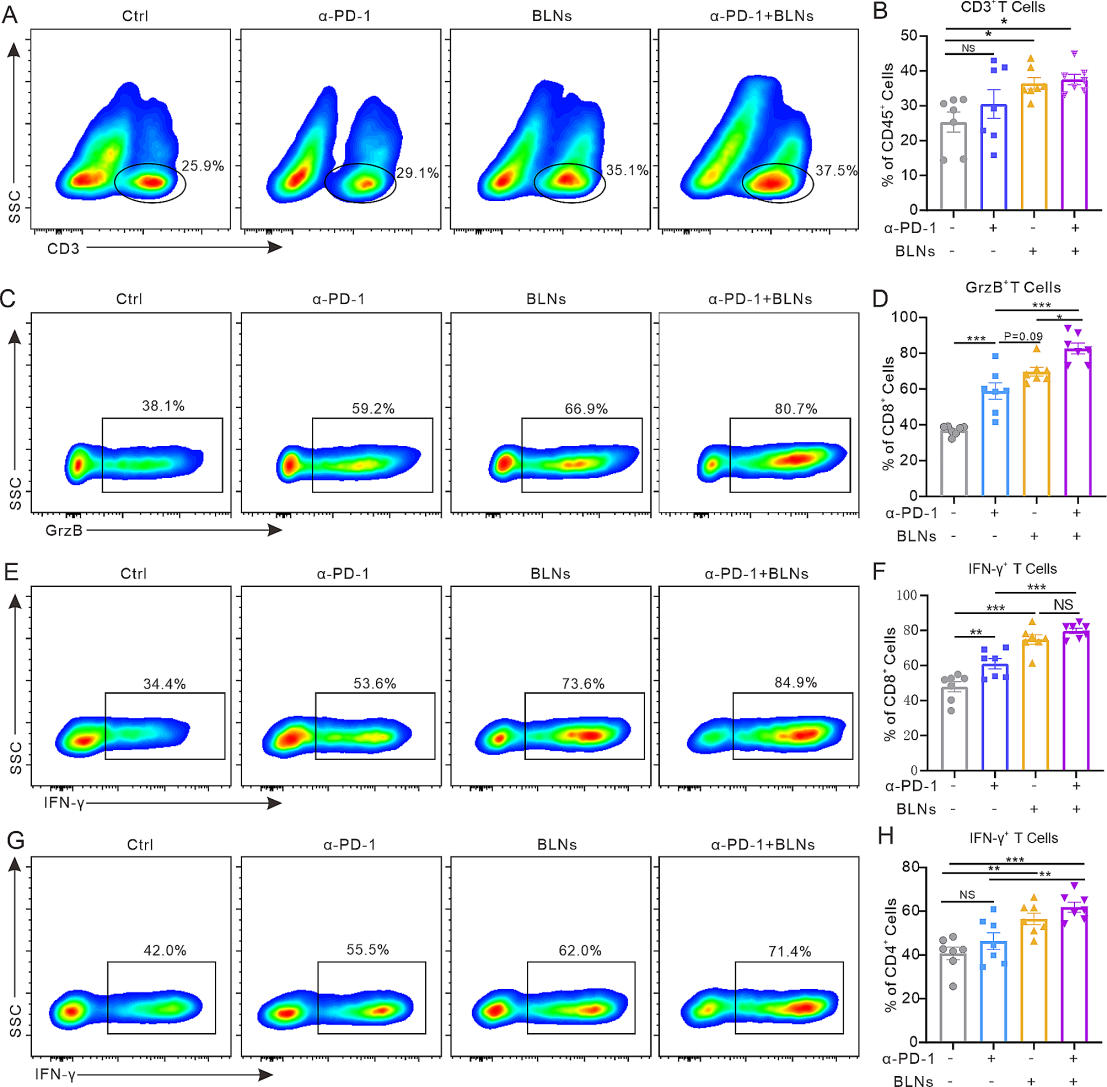
Combination of BLNs and PD-1 blockade significantly activates anti-tumor immunity. (**A, C, E, G**) Gating strategy to distinguish different immune cell types and representative results from the indicated treatment. (**B, D, F, H**) CD3^+^ T cell percentages among CD45^+^ cells, CD8^+^ GrzB^+^ T cell or CD8^+^ IFN-γ^+^ T cell percentages among CD8^+^ T cells, and CD4^+^ IFN‐γ^+^ T cell percentages among CD4^+^ T cells from the indicated treatments. Data are presented as the mean ± SEM (*n* = 5 to 7 per group)

BLNs activate tumoricidal immunity and promote anti-tumor immune cell infiltration. However, immune checkpoints such as PD-1 are major obstacles to T cell-mediated cancer cell killing. Therefore, we tested the capability of combining BLNs with α-PD-1 in modulating TME. Under combined treatment, the proportion and function of immune cells in the pleural perfusion fluid of MPE mice were largely altered. Flow cytometry analysis showed that the combination therapy led to more infiltration of CD3^+^ T cells than other groups (Fig. 5A, B). Furthermore, in CD3^+^ T cells, the percentage of CD8^+^ T cells increased and the percentage of CD4^+^ T cells decreased (Figure S5A, C, D). The increase in CD8^+^ GrzB^+^ T cells, CD8^+^ IFNγ^+^ T cells and CD4^+^ IFNγ^+^ T cells under combination therapy indicated activation of T cell-mediated tumor toxicity (Fig. 5C–H and Figure S5E). In the meanwhile, the level of Treg (Foxp3^+^ CD4^+^ T cells) was not significantly changed (Figure S5B, F). These results suggest BLNs combined with α-PD-1 promote infiltration and function of anti-tumor T cells.

### CD8^+^ T cells and macrophages dominate the combination therapy efficacy

**Fig. 6:**
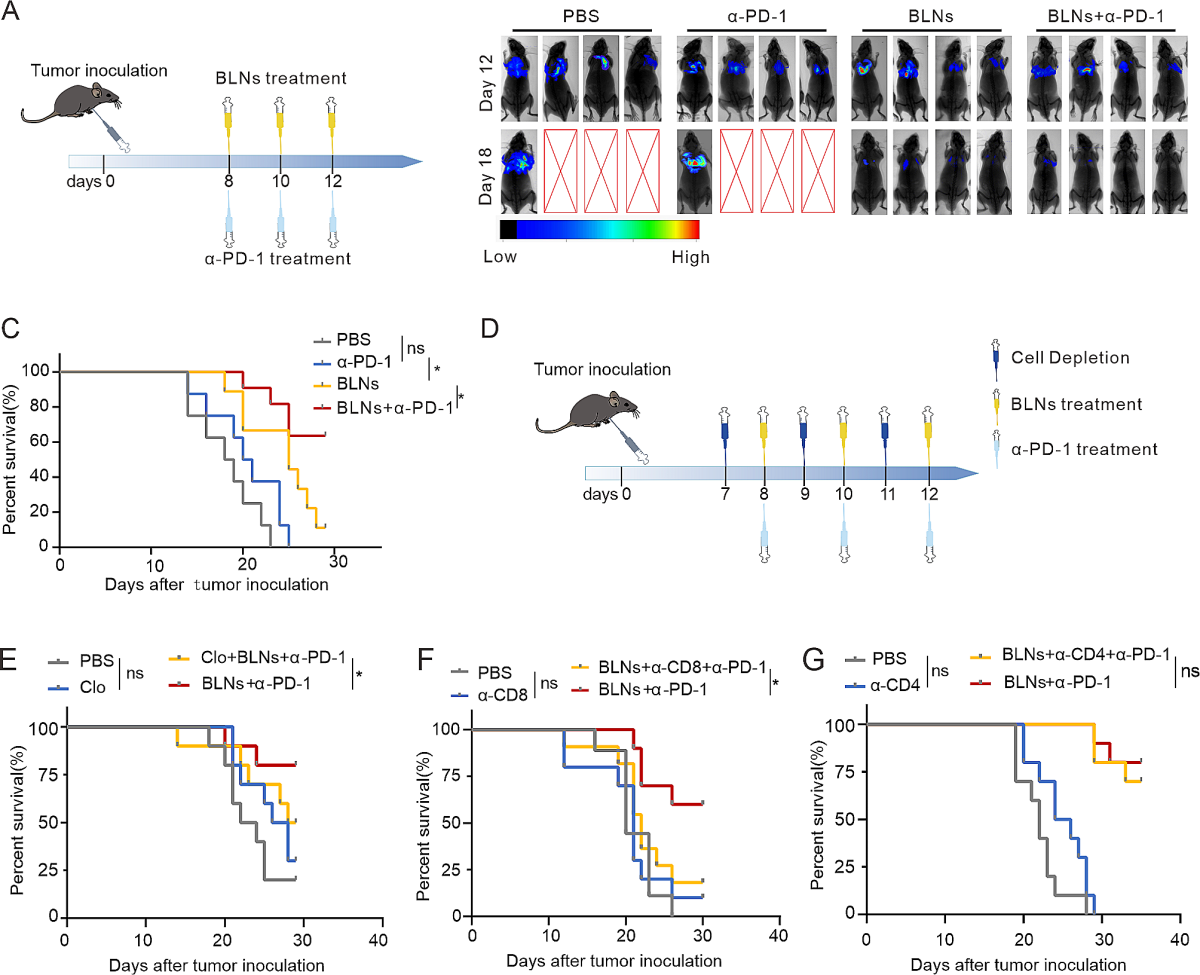
CD8^+^T cells and macrophages dominate the combination therapy efficacy. (**A**) Experimental outline of model establishment time and drug injection time. (**B**) Representative in vivo bioluminescence images showing the growth of mice MPE after various treatments. (**C**) Survival statistics for different treatment groups in MPE mice inoculated with LLC (*n* = 10). (**D**) Experimental outline of treatment and cell depletion. (**E**) Survival statistics of LLC-Luc MPE-bearing C57BL/6 mice (*n* = 9 to 10 per group) that were treated with clodronate liposomes and/or BLNs plus anti-PD-1. **(F, G) **Survival statistics of LLC-LUC MPE-bearing C57BL/6 mice (*n* = 9 to 10 per group) that were treated with anti-CD8 (**F**) or anti-CD4 (**G**) neutralizing antibody and/or BLNs plus anti-PD- 1

Three doses of BLNs combined with α-PD-1 were administrated every other day beginning on day 8 after tumor inoculation (Fig. 6A). In vivo imaging demonstrated that the combination therapy effectively delayed the progression of MPE (Fig. 6B). In mice inoculated with LLC or B16-F10, the combination of BLN and α-PD-1 demonstrated a notably higher efficacy in improving survival rates, as compared to the use of BLN or α-PD-1 alone. The survival rate could potentially reach as high as 63.6%. (Fig. 6C, Figure S6A). We then investigated the role of macrophages, CD4^+^ T cells, and CD8^+^ T cells in the combination therapy of MPE. This was achieved by effectively depleting macrophages, CD4^+^ T cells, and CD8^+^ T cells using Clodronate liposome (Clo), anti-CD4 antibodies, and anti-CD8 antibodies, respectively (Figure S6B-G). Depletion of CD8^+^ T cells significantly weakened the therapeutic effect of the combination therapy (Fig. 6E), which was followed by clearing macrophages (Fig. 6F). However, clearance of CD4^+^ T cells had a negligible effect (Fig. 6G). This indicates the dominance of CD8^+^ T cells and the auxiliary role of macrophages in the combination regime. In conclusion, these findings suggest that combining BLNs with α-PD-1 therapy initiates a tumoricidal immunity which is dependent on CD8^+^ T cells and macrophages.

### BLNs generate effective immune memory with secure biocompatibility

**Fig. 7:**
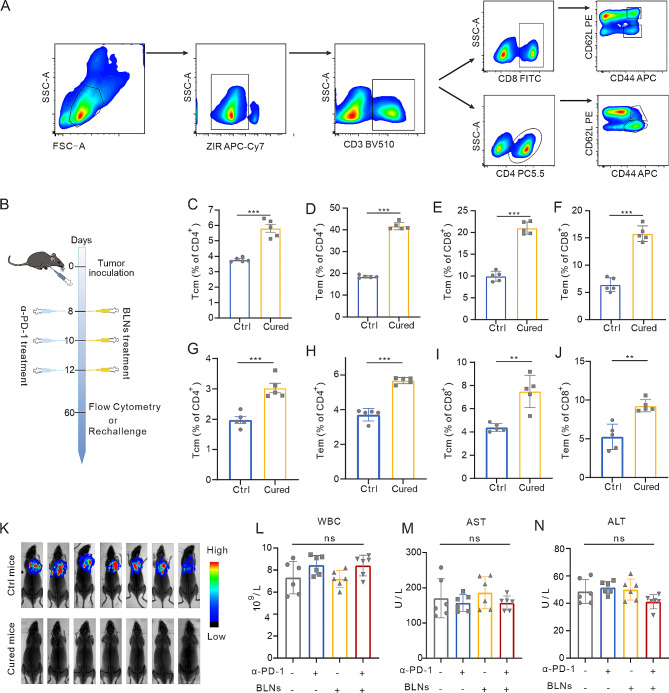
BLNs generate effective immune memory with secure biocompatibility. (**A**) Gating strategy to distinguish different immune memory cell types in MPE mice cured by combination therapy. (**B**) Experimental outline of mouse MPE immune memory model establishment time and drug injection time. (**C-J**) Flow cytometry analysis showed that CD4^+^ Tcm, CD4^+^ Tem, CD8^+^ Tcm and CD8^+^ Tem in the spleen (**C-F**) or lymph node (**G-J**) of MPE mice cured by combined therapy were significantly higher than those of control group. (*n* = 5 per group) (**K**) Representative in vivo bioluminescence images to monitor the growth of rechallenged thorax-injected LLC-LUC tumors. (*n* = 7 per group). (**L-N**) Hemanalysis was performed on the peripheral blood from mice on day 3 after treatment

Formation of tumor-antigen-recognition immune memory determines efficacy of treatments dependent on effectively activating anti-tumor immunity to inhibit tumor progression over the long-term [25]. Therefore, we investigated whether combining BLNs with α-PD-1 therapy can promote the formation of immune memory. In this study, CD3^+^CD4/CD8^+^CD44^high^CD62L^high^ T cells were gated as central memory T Cells (Tcm) and CD3^+^CD4/CD8^+^CD44^high^CD62L^low^ T cells were gated as effective memory T Cells (Tem) (Fig. 7A) [26]. After the last administration of combined therapy, all mice were kept till Day 60 to examine immune memory formation test (Fig. 7B). Both Tcm and Tem in CD4^+^ T cells or CD8^+^ T cells from spleen (Fig. 7C-F) or lymph node (Fig. 7G-J) significantly increased. To further validate the immune memory effect in vivo, we rechallenged five cured MPE mice with LLC-Luc. Immature mice of the same age were used as parallel controls. All the immature mice developed MPE, while the cured mice showed no indications of malignancy (Fig. 7K). Besides, we used the spleen grinding fluid from cured MPE mice to treat LLC. LDH detection revealed that the killing effect of the spleen grinding fluid on tumor cells was stronger in the cured group than in the control group (Figure S7A). Therefore, in general, BLNs combined with α-PD-1 can promote the formation of anti-tumor immune memory.

To assess the biocompatibility and safety of combining BLNs with α-PD-1, we collected blood samples for regular and biochemical analyses and collected important organ tissues (heart, liver, spleen, lung, and kidney) for histopathological staining. Both single and combination therapy did not impact the levels of white blood cells (WBCs), alanine aminotransferase (ALT), or aspartate aminotransferase (AST) (Fig. 7L-N). Histopathological examination revealed that these organs were normal and not affected by either single or combination therapy (Figure S8A). Together, these data demonstrate that BLNs elicit durable immune memory to fight against malignancy progression with reliable biosafety.

## Discussion

Although cancer immunotherapy, such as ICIs, has achieved great success in the field of cancer treatment, the magnitude of the benefit is highly variable [27]. Resistance to ICIs is partly associated with the underlying immunological characteristics of the TME, which is typically categorized as a “hot” (inflamed) or “cold” (non-inflamed) phenotype. The inflamed TME is characterized by the infiltration of lymphocytes and specifically the presence of an abundance of tumor-infiltrating lymphocytes (TILs) [28]. However, even the inflamed TME shows poor responses to ICIs [29]. In addition to the immunosuppressive TME, cancer cells intrinsically down-regulate the expression of immune sensors to evade immunological surveillance [30]. Given the above two reasons, we fabricated the BLNs and proved that BLNs show profound anti-cancer activity in vivo and in vitro. What was more, BLNs reversed the resistance to ICIs of MPE, and realized effective long-term memory protection to prevent cancer recurrence.

As one of the mainstays of first-line treatment in various solid cancers, RT has profound immunostimulatory effects [31]. Studies have shown that RT is a promising combination partner with ICIs and other immuno-oncology agents [32–34]. However, RT is usually recognized as a local treatment for cancer. It is of great value to apply the effects of RT to metastatic cancers. In a previous study, we found that RT-MPs can mimic the effects of RT by inducing cancer cell immunogenic cell death and reprogramming the phenotype of TAMs. Similarly, derived from irradiated-cancer cells, BLNs show stronger anti-cancer activity than RT-MPs in vitro and in vivo. This may be partly due to the fact that BLNs induce more CRT expression on the cell membrane as well as the release of soluble immunogenic mediators, such as calreticulin, ATP, and HMGB1.

TAMs and their precursors account for the largest fraction of the myeloid infiltrate in human solid cancers. The TAMs group is highly dynamic and heterogeneous, and most of them are immunosuppressive and support cancer progression, ultimately correlating with poor disease outcomes [35, 36]. Though a variety of small molecules and monoclonal antibodies (mAbs) targeted CSF1R or its ligand CSF1 are developed to reduce the infiltration of TAMs in the TME, the therapeutic efficacy is not satisfactory in clinical trials [37, 38]. Instead of removing TAMs from the TME, reprogramming TAMs to an anti-cancer phenotype is another attractive strategy, and has shown to be effective in preclinical models [39, 40]. Cytokines, chemo-inhibitors and other finely designed nanomaterials pave the way for this promising strategy [41, 42]. In a study of Wang et al., they developed pH-sensitive polymers containing IL-12, which reprograms TAMs to M1 macrophages [43]. Furthermore, Carbohydrate-containing nanomaterials were found to repolarize TAMs to M1 macrophages through upregulation of IL-12 and decrease classic M2 markers [44]. Finally, mitogen-activated protein kinase (MEK) and histone deacetylase (HDAC) inhibitors were found to suppress M2 polarization to hinder tumor progression [45]. In our study, BLNs engulfed TAMs showed an M1-like phenotype in vivo and in vitro, boosting potent anti-cancer immune response. These results showed that BLNs can be used as an alternative new nano-drug for the functional remodeling of TAMs for better cancer immunotherapy.

**Fig. 8:**
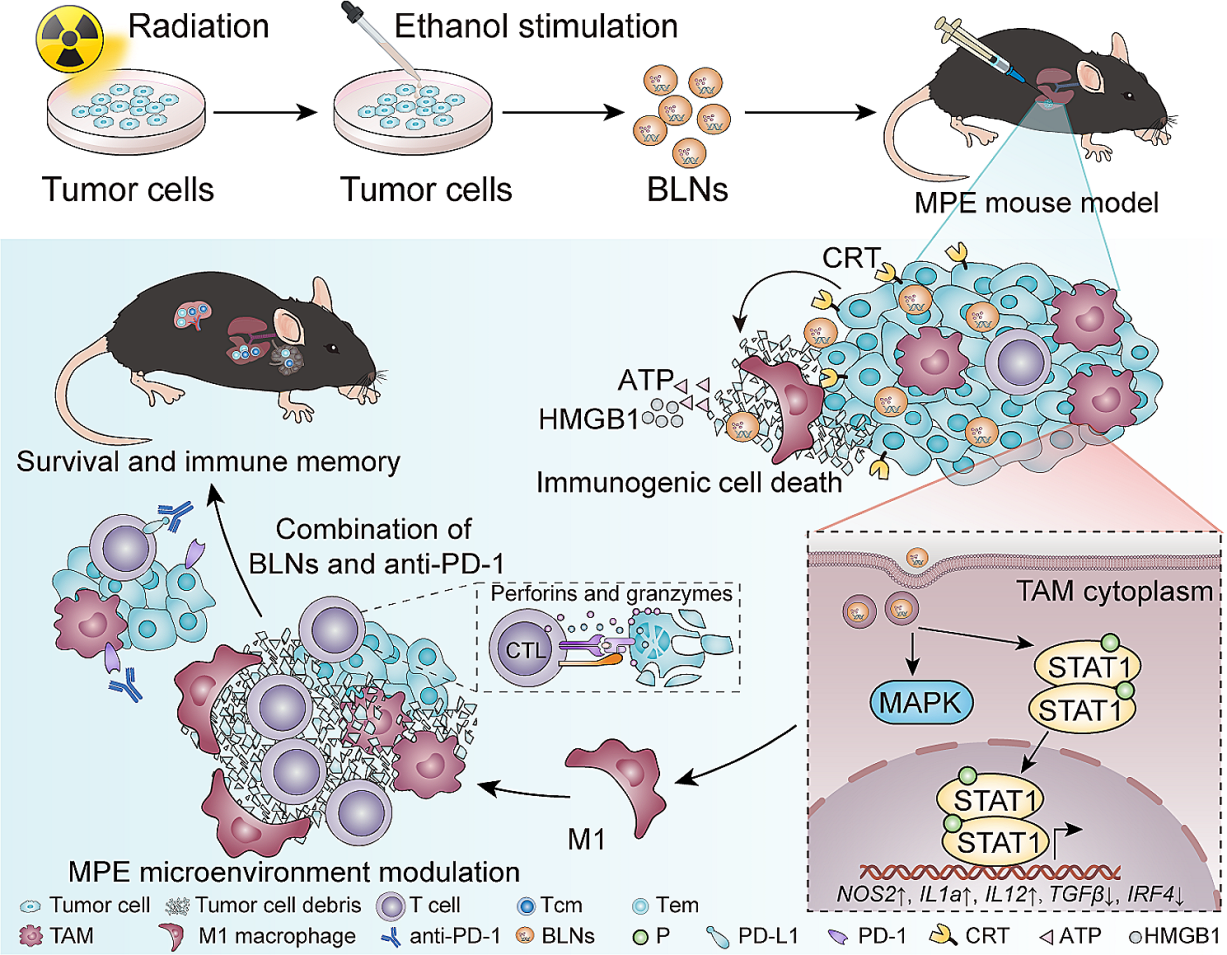
The schematic diagram of the mechanisms explored in this study

In conclusion, we described the development and biological function of a novel irradiated-cancer cell-derived BLNs (Fig. 8). The key findings of our study are that the BLNs (1) have good biological properties, and can easily be engulfed by cancer cells and TAMs, (2) remodel both the immunogenicity of cancer cells and phenotype of TAMs, which are the key mechanisms by which BLNs boost the anti-cancer immune response and reverse the resistant of MPE to α-PD-1, (3) have a synergistic anti-cancer effect with α-PD-1, leading to a completely eradication of cancer cells in the MPE mouse model and immunological memory effects were induced. (4) have good biocompatibility, and confers a chemo-free cancer treatment, which is of high security. In addition, we found that BLNs can mimic the biological effect of RT, providing a strategy to expand the clinical indications of RT, thus comprising indirect cancer RT to cancers where RT cannot be applied. The current study, therefore, presents a unique strategy for reversing immunotherapy resistance.

## Materials and methods

### Mice

C57BL/6 female mice were obtained from the Hunan Slyke Jingda Laboratory Animal Co. LTD. All the mice were bred and maintained in a specific pathogen-free (SPF) barrier facility in the Animal Center of Huazhong University of Science and Technology (HUST; Wuhan, China). All animal studies were approved by the Hubei Provincial Animal Care and Use Committee and followed the experimental guidelines of the Animal Experimentation Ethics Committee of the Huazhong University of Science and Technology.

### Cell lines and cell culture

Mouse LLC, B16-F10 cells were obtained from the China Center for Type Culture Collection (Wuhan, China). The luciferase stably transfected cell lines (LLC-Luc and B16-F10-Luc) and RFP stably transfected cell lines (LLC-RFP) were established in the lab. Cells were grown in Dulbecco’s Modified Eagle’s Medium (DMEM) or RPMI 1640 medium (Gibco, Grand Island, NY, USA) containing 10% Fetal Bovine Serum (FBS) (Gibco, Grand Island, NY, USA) and 1% penicillin/streptomycin solution.

### Isolation of BLNs and RT-MP

A total of 5 × 10^6^ cells that were plated into 10-cm cell culture dishes were irradiated with a single dose of 10 Gy by 6-MV x-rays (600 MU/min, Trilogy System Linear Accelerator, Varian Medical Systems). The medium was then replaced with 20 mL of complete medium (DMEM or RPMI 1640, based on the needs of each cell line). After 72 h, 30% ethanol was added for a 10-minute stimulation, after which medium was collected and centrifuged at 1000 g for 10 min, followed by centrifugation at 14,000 g for 2 min to remove tumor cells and debris. Then, supernatants were centrifuged at 14,000 g for 1 h at 4 °C to isolate BLNs and further centrifuged at 120,000 g for 70 min at 4 °C to isolate exosomes. The pellet (containing exosomes or MPs) was washed twice with sterile 1×PBS and resuspended in sterile 1×PBS for animal experiments or resuspended in intact medium for cell experiments.

### Transmission electron microscopy

BLNs were observed by TEM. BLNs in suspension were stained with 2% phosphotungstic acid solution for 5 min and then deposited on copper mesh. Size and morphology were observed by TEM (HT7700-SS/FEI Tecnai G20 TWIN).

*Western blotting* Cells or EVs were treated with RIPA lysis buffer and protein separation was performed on 8% SDS-PAGE gel. After a 1-hour blocking step with 0.5% skim milk powder/tris-buffered saline (TBS)-5% tween (TBS-Tween), membranes were incubated overnight at 4 ºC with the following primary antibodies: CD9 antibody (EPR2949, Abcam), TOM20(66777-1-Ig, Proteintech), HSP60(66041-1-Ig, Proteintech), Histone H3(68345-1-Ig, Proteintech), TIMM23 (Tyr185) Recombinant antibody (80,024‐1‐RR, Proteintech), Phospho‐ERK1/2 (Thr202/Tyr204) Polyclonal antibody (28,733‐1‐AP, Proteintech), Phospho-STAT1 (Tyr701) Polyclonal Antibody (44-376G, Invitrogen) and Phospho‐p38 MAPK (Thr180/Tyr182) Polyclonal antibody (28,796‐1‐AP, Proteintech). All images were acquired using the ChemiDoc Imaging System (Bio‐Rad).

### Generation of BMDMs

The femoral specimens of C57BL/6 male mice aged from 6 to 12 weeks were collected. Red blood cells were first depleted with red blood cell lysis buffer and then differentiated in RPMI 1640 medium supplemented with 10% FBS and macrophage Colony-stimulating factor (20 ng ML; PeproTech). Change the media every two days. On Day 6, naive macrophages (BMDMs) were stimulated with IL-4(20 ng/ml; PeproTech) for 24 h to generate BMDM-M2 macrophages. The BMDM-M2 cells were collected on the 7th day for the follow-up test.

### In Vitro Cellular Uptake Assay

To determine the cellular colocalization of BLNS with BMDMs, BMDMs were seeded in a glass-bottom cell culture dish (NEST, cat. No. 801,001; 1 × 105 per well) and incubated with PKH26-labeled BLNs for 2, 6, 12, and 24 h. Subsequently, these cells were washed three times in PBS and then stained with carboxyfluorescein diacetate succinimidyl ester (10 μm) for 10 min. After that, the cells were washed with PBS and fixed in 4% paraformaldehyde for 30 min before the cells were washed with PBS. Cells were imaged using Confocal laser scanning microscopy imaging (Olympus FV3000). To quantitatively assess cell uptake, cells were seeded in six-well cell dishes and treated as above, then washed three times in PBS, collected, fixed, and resuspended in PBS (150 ΜL) for Flow cytometry detection.

### ATP release assays in vitro

To measure extracellular ATP levels, cell culture supernatants were collected and ATP concentrations were measured using the luciferin-based ENLITEN ATP assay (Promega) kit according to the instructions.

### Model animal experiments and evaluation of therapeutic effects

Mice used in the experiment were matched for age (6 weeks), weight (18 to 20 g) and sex. To establish a model of MPE, mice were anesthetized with 1% pentobarbital prior to all operations. LLC-LUC cells were injected into the right thoracic cavity through the 10th or 11th intercostal space of the midaxillary line. Seven days after LLC-LUC cell inoculation, each mouse was observed by bioluminescence imaging to ensure successful and uniform establishment of MPE model. Then the mice were randomly divided into control group, BLNS group, α-PD-1 group, BLNS combined with anti-PD-1 group, and treated. Mice were anesthetized with isoflurane and then intrapleural injection of 50µL fluids (PBS or BLNs suspension) or by intraperitoneal injection of α-PD-1 (10 mg/kg), both treated every other day. To assess MPE growth, six mice in each group were imaged on the day all treatments were completed using Bruker in vivo MS FX Pro Imager under 1% pentobarbital anesthesia.

### T cell depletion

Anti-mouse CD4 monoclonal antibody (clone GK1.5) or anti-mouse CD8 monoclonal antibody (clone 2.43) were used to deplete CD4^+^ T lymphocyte or CD8^+^ T lymphocyte in mice. On the day before treatment, mice were intraperitoneally injected with antibodies at a dose of 200 µg/mouse, once every two days for a cumulative period of three times, followed by two doses of 100 µg/mouse.

### Macrophage depletion

Clodronate liposomes (FormuMax, F70101C-AC) were used to deplete macrophages in mice. The mice were injected intraperitoneally with clodronate liposomes at a dose of 200 µl/mouse 1 mL one day before treatment, once every two days for a cumulative period of three doses, followed by two doses of 150 µg/mouse.

### Flow Cytometry

Cells were stained with the anti-mouse Zombie NIR reparable survival kit (423,106) and incubated with anti-CD45(103,114), CD11B (101,205), f 4/80(123,121), CD3(100,212), CD4(100,408), and CD8A (100,752) at recommended concentrations for 30 min at 4 ° C to stain the cell surface. For intracellular IFN-γ (505,808) cytokine staining in T cells, phorbol 12-myristate 13-acetate (PMA) was used first (AB120297, ABCAM, 100 ng/mL), monensin sodium salt (AB120499, Abcam, 1 µg/mL) and ionomycin calcium salt (5,608,212, PeproTech, 100 ng/mL) stimulated T cells for 6 h, after which cells were fixed and permeabilized. For CD206(141,706) staining, cells were also immobilized and permeabilized. All Flow cytometry antibodies were purchased from BioLegend (San Diego, CA).

### Immunofluorescence

Cells were fixed with 10% paraformaldehyde PBS, incubated with 1% Triton X-100 PBS for 10 min and blocked with 3% BSA PBS, and then stained for BMDMs with mouse anti-F4/80 antibody (ab6640, Abcam). Nuclei were stained with 4,6-diamidino-2-phenylindole. Use the Fluorescence microscope (Olympus FV3000) for detecting.

*Bioluminescence Imaging* After Lewis MPE mice had been anesthetized with 1% pentobarbital sodium, they were intraperitoneally injected with firefly luciferin (150 mg/kg; Sigma-Aldrich; CAS: 103,404‐75‐7). After 15 min, mice were imaged using the Bruker In Vivo MS FX PRO Imager. The luminescent images were acquired with 3‐min exposure times, and X‐ray photographs were taken with 30 s exposure times.

### Real-time quantitative polymerase chain reaction

RNA extraction of LLC cells was performed using the Total RNA Kit I R6834 (Omega), and was measured using the NanoDrop ND-1000 (Thermo Fisher Scientific). Purified RNA was reverse-transcribed into complementary DNA by qPCR (+ gDNA wiper) using the HiScript III RT SuperMix (Vazyme), according to the manufacturer’s protocol. RT-PCR reactions were performed in the Step One system using ACEQ Universal SYBR QPCR Master Mix (Vazyme).

### RNA sequencing

BMDMs were treated with or without BLN for 24 h, after which they were washed twice with PBS, centrifuged at 1000 g for 10 min, and supernatants were discarded. The cells were rapidly frozen in Trizol reagent at -80 °C. Then, samples were sent to Beijing Novogene Technology Co., Ltd for RNA sequencing.

### Lactate dehydrogenase assay

The Lactate dehydrogenase assay kit (Applygen Technologies Inc, Beijing, China) was used to determine Lactate dehydrogenase (LDH) levels in cells. Splenocytes were harvested from cured mice and single-cell suspensions were prepared by homogenizing them using ground glass slides. Splenocytes (4 × 10^7^) were cultured with UV-irradiated LLC tumor cells. The cells were harvested five days later, and used as CTL effector cells in a standard lactate dehydrogenase cytotoxicity assay, in which LLC tumor cell targets were seeded at 10,000 cells per well. The percentage of specific killing was defined as (experimental value- effector cells spontaneous control- target cells spontaneous control)/ (target cell maximum control- target cells spontaneous control) × 100%.

### Statistical analysis

Unpaired two-tailed Student’s t test was used to compare differences between the two groups, whereas survival was assessed using the log-rank Mantel-cox test of Graphpad Prism 9 software. Repeated measures analysis of variance was used to compare tumor volume growth with one-way analysis of variance (ANOVA). Use FlowJo to analyze Flow cytometry data. Significant differences between groups were indicated by * *p* < 0.05, * * *p* < 0.01, * * * *p* < 0.001.

### Electronic supplementary material

Below is the link to the electronic supplementary material.

Supplementary Material 1

## Data Availability

The data that support the findings of this study are available from the corresponding authors upon reasonable request.
